# Acute Treatment With Gleevec Does Not Promote Early Vascular Recovery Following Intracerebral Hemorrhage in Adult Male Rats

**DOI:** 10.3389/fnins.2020.00046

**Published:** 2020-02-04

**Authors:** Mohammed Abbas, Elizabeth Haddad, Mary Hamer, Derek Nowrangi, John Zhang, William J. Pearce, Jiping Tang, Andre Obenaus

**Affiliations:** ^1^Department of Pediatrics, Loma Linda University, Loma Linda, CA, United States; ^2^Department of Pediatrics, University of California, Irvine, Irvine, CA, United States; ^3^Department of Physiology and Pharmacology, Loma Linda University, Loma Linda, CA, United States; ^4^Department of Anesthesiology, Loma Linda University, Loma Linda, CA, United States; ^5^Department of Neurosurgery, Loma Linda University, Loma Linda, CA, United States; ^6^Center for Perinatal Biology, Loma Linda University, Loma Linda, CA, United States

**Keywords:** cerebrovasculature, vessels, vessel painting, magnetic resonance imaging, edema, fractal

## Abstract

Intracerebral hemorrhage (ICH) remains one of the most debilitating types of stroke and is characterized by a sudden bleeding from a ruptured blood vessel. ICH often results in high mortality and in survivors, permanent disability. Most studies have focused on neuroprotective strategies designed to minimize secondary consequences and prevent further pathology. Lacking is an understanding of how ICH acutely affects cerebrovascular components and their response to therapeutic interventions. We hypothesized that ICH alters cortical vessel complexity in the parenchyma adjacent to site of the initial vascular disruption and that vascular abnormalities would be mitigated by administration of the PDGFR inhibitor, Imatinib mesylate (Gleevec). Briefly, ICH was induced in male adult rats by injection of collagenase into basal ganglia, followed by Gleevec administration (60 mg/kg) 1 h after injury. Rats were then perfused using vessel painting methodology (Salehi et al., 2018b) to stain whole brain vascular networks at 1 day post-ICH. Axial and coronal wide field fluorescence microscopy was performed. Analyses for vascular features were undertaken and fractal analysis for vascular complexity. Data were collected from four groups of rats: Sham + Vehicle; Sham + Gleevec; ICH + Vehicle; ICH + Gleevec. Microscopy revealed that cortical vessels in both ipsi- and contralateral hemispheres exhibited significantly reduced density and branching by 22 and 34%, respectively. Fractal measures confirmed reduced complexity as well. Gleevec treatment further reduced vascular parameters, including reductions in vessel density in tissues adjacent to the ICH. The reductions in brain wide vascular networks after Gleevec in the current study after ICH is contrasted by previous reports of improved behavioral outcomes and decreased lCH lesion volumes Reductions in the vascular network after Gleevec may be involved in long-term repair mechanisms by pruning injured vessels to ultimately promote new vessel growth.

## Introduction

Spontaneous intracerebral hemorrhage (ICH) is defined as a sudden bleeding from ruptured cerebral blood vessels. It accounts for about 10–20% of all strokes (Feigin et al., 2009) and is associated with higher mortality and morbidity leading often to worse outcomes compared to other strokes (Broderick et al., 1999). ICH occurs primarily when a hematoma rapidly accumulates within brain parenchyma (Aronowski and Zhao, 2011) with a persistent expansion over 6–24 h (Aguilar and Brott, 2011). This leads to further mechanical disruption of the normal parenchymal anatomy (Aronowski and Zhao, 2011) followed by subsequent tissue compression effects and increased intracranial pressure (Qureshi et al., 2009). This expansion of the hematoma is highly predictive of neurological deterioration, poor functional outcome and mortality (Broderick et al., 1993; Kazui et al., 1996, 1997; Leira et al., 2004). The hemorrhagic expansion can be attributed to leakage or rebleeding from secondary mechanical shearing of vessels surrounding hematoma (Fisher, 1971) but other cellular processes are also known to contribute including, but not limited to: (a) initiation by thrombin of the coagulation cascade leading to activated microglia (Wagner et al., 2002; Nakamura et al., 2005; Nakamura et al., 2006; Xi et al., 2006), (b) hematoma breakdown-products such as hemoglobin (Mracsko and Veltkamp, 2014), and (c) a robust inflammatory response (Wang and Dore, 2007; Yang et al., 2016). These secondary outcomes lead to a disrupted BBB resulting in significant brain edema (Hua et al., 2007) that are strongly predictive of functional outcomes (Gebel et al., 2002). While neuroinflammation and other secondary injury cascades in the context of ICH have been well studied previously (Yang et al., 2016), little has been reported on the how ICH modifies cerebrovascular integrity and morphology. We have previously reported that ICH results in a modified cerebral vascular smooth muscle phenotype (Pearce et al., 2016). However, the morphological features of the injured cerebrovasculature including integrity of the neurovascular unit are critical features that are poorly understood. Clinically, the microvascular integrity has been cited as a potential key factor in hematoma expansion in one third of ICH patients (Chen et al., 2017). Thus, a more complete understanding of how the vasculature is altered following ICH is warranted.

To our knowledge, there are no comprehensive studies examining the vascular morphology of parenchymal vessels following ICH nor the vasoprotective effects Gleevec. Thus, we set out to investigate the microvascular morphological changes adjacent to perihematomal parenchyma and in cortical regions remote from the site of the ICH lesion. To examine whole brain vessel morphology, we applied our novel vessel painting technique as we have reported in other models of acquired brain injury (Obenaus et al., 2017; Salehi et al., 2018b). This approach allows whole brain staining of the vasculature and is amenable to confocal microscopy for derivation of vascular morphological details. In the present study we first investigated how ICH modifies vascular morphological features followed by assessment of how Gleevec treatment could maintain vascular morphology acutely after ICH. We examined the vascular plexus in cortex and basal ganglia closely and analyzed their features in both hemispheres.

Gleevec or Imatinib Mesylate was developed to treat chronic myelogenous leukemia (CML) and is known to have multiple roles (Soverini et al., 2018). Gleevec has been shown to minimize stroke damage by its actions on the vasculature (Su et al., 2008). More recently we and others have identified that Gleevec antagonism of the platelet-derived growth factor receptor-β (PDGFR-β) on vascular elements. We reported that vascular smooth muscle phenotype is modified after ICH and that Gleevec mitigates this phenotypic switch (Pearce et al., 2016; Yang et al., 2016). Others have also shown that PDGF and its family play an important role in vascular protection using the retina as a model system (He et al., 2014). We investigated the role of Gleevec (Imatinib), a PDGFR-β antagonist, on vessels and how it influenced vascular measures acutely (1 day) after ICH. Thus, this study reports that ICH induces vascular loss, locally at the site of ICH and in regions distant from the injury. Moreover, acute assessment of Gleevec treatment found no significant protection of the cerebrovasculature during the acute assessment period.

## Materials and Methods

### Animals

All animal experiments and care were in compliance with federal regulations and approved by the Loma Linda University Institutional Animal Care and Use Committee. Thirty male Sprague Dawley rats weighing 250–300 g (2–5 months old) were housed in a temperature controlled animal facility on a 12 h light-dark cycle and were allowed free access to food and water. Animals were randomly assigned to experimental groups. Aseptic techniques were used for all surgeries. No rats died or were excluded from the study due adverse outcomes from intracerebral hemorrhage injury (ICH).

### Experimental Groups

All drug administration was performed by within 1 h following ICH induction, where vehicle (PBS and 0.1% DMSO) or 60 mg/kg Gleevec were injected intraperitoneally. Rats were subdivided randomly into four groups (*n* = 6/group): (a) Sham + vehicle, (b) sham + Gleevec, (c) ICH + vehicle, (d) ICH + Gleevec. Neurological function and behavior were assessed 24 h after ICH induction.

### Intracerebral Hemorrhage Injury (ICH) Model

ICH was induced using the Bacterial Collagenase injection model as previously described (Pearce et al., 2016). Rats underwent isoflurane anesthesia (4% induction with 2% maintenance in 70% medical air and 30% O_2_) and then were secured prone onto a stereotactic frame (David Kopf Instruments, Tujunga, CA, United States). Body temperature was maintained at 37.0°C ± 0.5°C using a thermostat-controlled heating blanket throughout the entire surgical procedure. The following stereotactic coordinates were used to localize the right basal ganglia: 0.2 mm anterior, 5.6 mm ventral, and 2.9 mm lateral to the bregma. An incision was then made over the scalp and a posterior cranial burr hole (1 mm) was drilled over the right cerebral hemisphere into which a 27-gauge needle was inserted at a rate of 1 mm/min. A micro infusion pump (Harvard Apparatus, Holliston, MA, United States) infused the bacterial collagenase Type VII-S (0.2 U/μL; Sigma-Aldrich, St. Louis, MO, United States) through a Hamilton syringe at a rate of 0.2 μL/min. The needle remained in place for 10 min to prevent back-leakage before being slowly withdrawn (1 mm/min). The borehole was then sealed with bone wax, the incision sutured closed and the animals were monitored during the recovery period. Sham animals received the identical manipulations but instead of collagenase injection they received vehicle infusion. Food, water, and post-operative supportive care were provided, as is routinely done. Animals were euthanized 24 h after surgery for assessment of vascular morphology.

### Vessel Painting and Tissue Fixation

**FIGURE 1:**
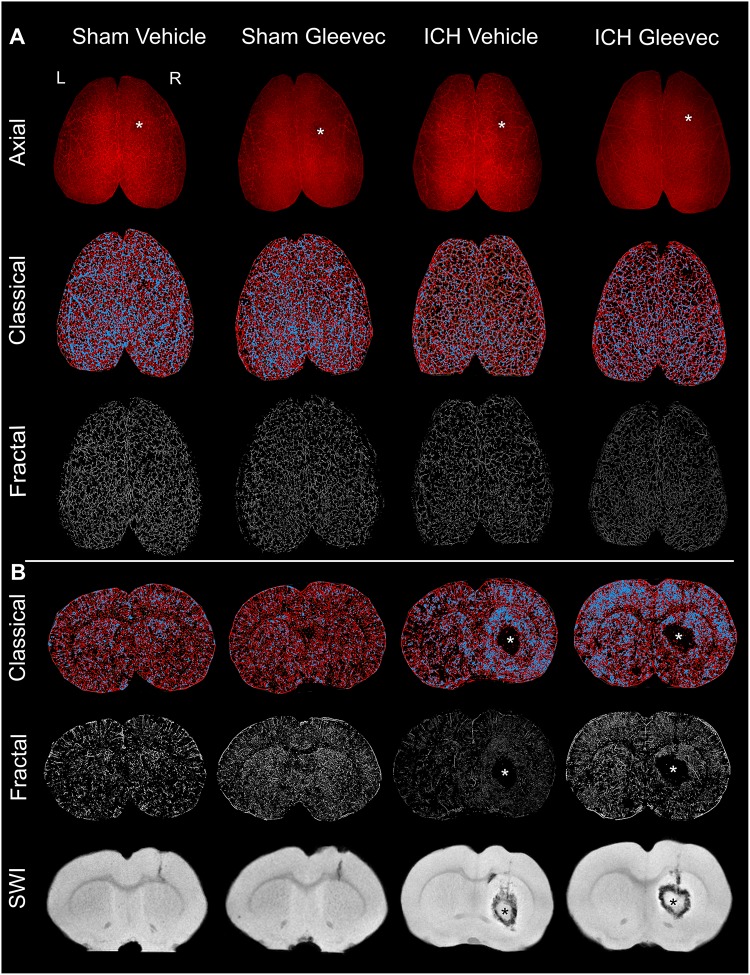
Intracerebral hemorrhage (ICH) results in cerebral vascular decrements at 24 h after injury or treatment. **(A)** Axial: The top row illustrates representative axial vessel painted whole brains in ICH animals with and without Gleevec treatment (* = injection site). The middle row are sample classical vascular analysis maps where red denotes vessels and blue demarcates vessel junctions. The third row displays fractal maps utilized for analysis of vascular complexity. **(B)** Coronal: Classical vascular analysis of coronal sections from all experimental groups wherein the ICH lesion in the striatum can be visualized in the injured rats (*). Middle row are the corresponding fractal analysis maps. The last row of coronal images are from susceptibility weighted magnetic resonance imaging (SWI MRI) which was utilized to monitor the extent of the hematoma and edema after ICH induction. Sham animals exhibited minimal vascular alterations from needle insertion whereas ICH animals have prominent and extensive areas of vessel disruption at the site of the ICH. All images in each column are from the same animal.

Animals were sacrificed via transcranial perfusion 24 h after ICH for vessel painting and for tissue fixation (Salehi et al., 2018b). Briefly, rats were anesthetized by intraperitoneal injection of ketamine (90 mg/kg) and xylazine (10 mg/kg), then perfused for vessel painting and finally tissue fixation. Our vessel painting protocol stains the vascular network using DiI (1,1′-dioctadecyl-3,3,3′,3′-tetramethylindocarbocyanine perchlorate) (Life Technologies, Carlsbad, CA, United States) which has been commonly used for labeling cell membranes (Schlessinger et al., 1977) and neuronal tissues (Honig and Hume, 1986; Godement et al., 1987). The lipophilic properties of DiI allow direct vascular staining. The perfusion device consisted of a 25- gauge butterfly needle and syringes for phosphate-buffered saline (PBS), DiI solution and Paraformaldehyde (PFA). After exposure of the heart a butterfly needle was inserted into the left ventricle. Heparin (0.02 mg/g, Sagent Pharmaceuticals) and sodium nitroprusside (0.075 mg/gm, Sigma-Aldrich, St. Louis, MO, United States) were injected followed by serial injections of 150 ml of PBS, 50 ml of DiI (13ug/ml) in diluent solution and 200 ml of 4% PFA. Care was taken to ensure that no air was trapped within the perfusion system. Brains were extracted from the cranium, rinsed in PFA and post-fixed for 24 h. Thereafter, vessel painted brains were stored in PBS until MRI or wide-field microscopy were undertaken. Individual photographs of vessel-stained brains were acquired to document the labeling efficacy. In this study, vessel painting was successful in 71% of animals wherein vascular staining was uniform (Figure 1).

### Magnetic Resonance Imaging Acquisition and Analysis

High resolution MRI was undertaken using an 11.7T Bruker Avance instrument (Bruker Biospin, Billerica, MA, United States) running with the Paravision software (version 5.1, Bruker Biospin) to assess brain injury volumes. T2-weighted images [T2WI; repetition time (TR)/echo time (TE): 2395 ms/10 ms, 20 × 1 mm slices] were acquired and collected on a 256 × 256 matrix with a 2 cm field of view to quantitate edema and extravascular blood.

Volumetric analysis of hematoma size and quantification of surrounding edema was performed on T2WI coronal slices using Cheshire image processing software (Hayden Image/Processing Group, Waltham, MA, United States, version 4.3). Investigators who were blind to treatment groups manually defined the region of interest (ROI) on each slice that included whole brain, hematoma (hypointensity), and edema (hyperintensity). Hematoma and edema area on each slice affected by the ICH injury were delineated based on the differentiation of hyper-/hypo- intensive regions of injury relative to surrounding normative tissue conditions. Any blood or edema on or below the corpus callosum was classified as subcortical injury, whereas any injury above the corpus callosum was classified as cortical injury. Whole brain analysis started where the olfactory bulb begins to merge with the cortex and spanned the entire cerebrum. To obtain edema volumes the hematoma volume was subtracted as the edematous region typically encased the hematoma. Cortical injury was analyzed separately and also combined with subcortical injury for total ICH injury volumes. All data were organized and summarized in Microsoft Excel.

### Vessel Painting Image Acquisition and Analyses

Vessel painted brains were imaged using Keyence BZ-9000 fluorescent microscope (Keyence BZ-X700; Keyence Corp., Osaka, Japan). Whole brains were gently positioned between 2 glass slides and lightly compressed to eliminate bubbles and improve visibility. Whole brain axial views were imaged at 2X with z-stacks using 60 μm steps with 50 slices resulting in 3 mm slab. After axial views were collected, the brains were bisected into 2 coronal segments with an anterior and posterior face at the level of the ICH lesion using MRI data as a guide. Coronal images were then scanned at the same resolution by gently fixing the anterior half of the brain on a single glass slide, examined at 2X, z-stacked at 60 μm with 30 slices resulting in 1.8 mm sections. Z-stacks for both axial and coronal were merged into full focus images using the BZ-II analyzer software (Keyence Corp., Version 1.2.0.1.). Finally, the software haze reduction feature (Blur/Brightness/Reduction: 10/10/1) was applied on the resultant images to remove any background and enhance the vascular network.

Classical vascular analysis was undertaken using the Image J plugin, Angiotool (RRID:SCR_016393) (Zudaire et al., 2011). Originally developed to assess spatial features of the angiogenic vasculature in the retina we have adapted this software for use in evaluating whole brain cortical vessels after traumatic brain injury (Obenaus et al., 2017; Salehi et al., 2018b). Angiotool features used in the current study were structural parameters such as vessel density and length, branch points and lacunarity (space without vessels).

Similar to our previous studies (Obenaus et al., 2017; Salehi et al., 2018b), we utilized fractal analysis to determine vascular complexity. Fractals have been widely used to describe structural complexity of various tissues, including the microvasculature of retina (Cheung et al., 2012), pituitary (Di Ieva et al., 2007), microglial complexity (Karperien et al., 2013) and cortical vessels (Obenaus et al., 2017). The ramified and irregular pattern of vessels after injury has made their evaluation by standard tools inaccurate and difficult. As we have done previously, we utilized the Fraclac ImageJ plugin (RRID:SCR_003070) to optimize quantification of vessels in cortex and basal ganglia (Schneider et al., 2012; Karperien et al., 2013). Briefly, binary images were analyzed by measuring the total number of pixels locally connected in a box of increasing size **ε** using the slope of the log regression line for pixel mass against scale (Karperien et al., 2013). The Local connected Fractal Dimension (DLC) is extracted and is calculated similar to the mass Fractal Dimension:

$$D_{B\,m\,a\,s\,s}=\lim_{\varepsilon \to 0}\left\lceil \text{ln}\,\left(\mu_{\varepsilon }\right)\,/\,\ln\left(\varepsilon \right)\right\rceil$$

Where με is the mean pixels per box at some size ε. The pixel mass is calculated from concentrically placed sampling units using a connected set at each pixel to produce a distribution of local variation in complexity in data as previously described (Obenaus et al., 2017).

We used ImageJ analysis software to convert vessel painted images into binary images. First, we outlined our area of interest on each image then we ran Fraclac DLC analysis for each brain and saved the results. The distribution of DLC (also denoted as local fractal dimension, LFD) for each brain was then further processed for kurtosis, skewness, peak fractal frequency and local fractal dimension (LFD) for group comparisons. The LFD represents patterns at different scales and is often used to represent complexity of the subject. Skewness and kurtosis of the fractal histograms offer additional measures on how uniformly the fractal dimensions were distributed and how (peaked) the fractal distribution appears, respectively. Analyzed images were colorized based on fractal dimension using a default LUT color coding.

### Laser Confocal Microscopy

Whole vessel painted brains and their coronal sections were imaged on the laser scanning confocal microscope Zeiss LSM 710 (Jena, Germany) to evaluate at high resolution vascular structures. Images were obtained (10X objective lens, excitation wavelength Abs: 549 nm, Ems: 565 nm) of the ipsilateral ICH hemisphere. Zeiss software (Zen) was used to process the z-stacks (28.4 μm steps) for resultant maximum intensity projections.

### Statistics

All data was tested for normal distribution and comparisons were made using *t*-test (unpaired). If samples did not have normal distributions data were evaluated using a Mann–Whitney test. All graphical and statistical analysis used GraphPad Prism 7 software (GraphPad Prism, San Diego, CA, United States; RRID: SCR_002798). All calculations were achieved without knowledge of group. All data are presented as mean ± SEM with statistical significance reported at *p* < 0.05.

## Results

The results are comprised of two components: (a) first we report the novel findings of how ICH impacts the cortical and basal ganglia vasculature following ICH, and (b) we then describe the results of how Gleevec treatment after ICH may modify the cortical and basal ganglia vessels. Representative axial and coronal vessel painted brains and the classical and fractal vessel analysis maps as well as magnetic resonance imaging (MRI) are illustrated in Figure 1. It is readily apparent that ICH results in vascular modifications.

### Vascular Modifications in the Cortex and Basal Ganglia 24 h After ICH

Visual examination of the wide field axial microscopic images of Sham + Vehicle (*n* = 9) and ICH + Vehicle (*n* = 9) vessel painted brains revealed a lack of overt gross cortical vascular changes (Figure 1A). Mild vascular disruptions at the injection sites on the cortex were visible in both Sham and ICH vessel painted brains (Figure 1A – denoted by ^∗^). However, in coronal images for classical vascular analysis maps, there was a visible apparent increase in junctional density after ICH, often adjacent to the hematoma (^∗^) (Figure 1B, blue dots). Quantitative analysis was undertaken to evaluate the effects of ICH on cortical and basal ganglia vessels. Multiple vascular morphological parameters were quantified including: (1) vessel density (% vessel/total area), (2) branching index (branch points or junctions/unit area), (3) total vessel length, and (4) lacunarity, which describe areas that do not have vascular elements.

#### Axial Cortex Analysis

**FIGURE 2:**
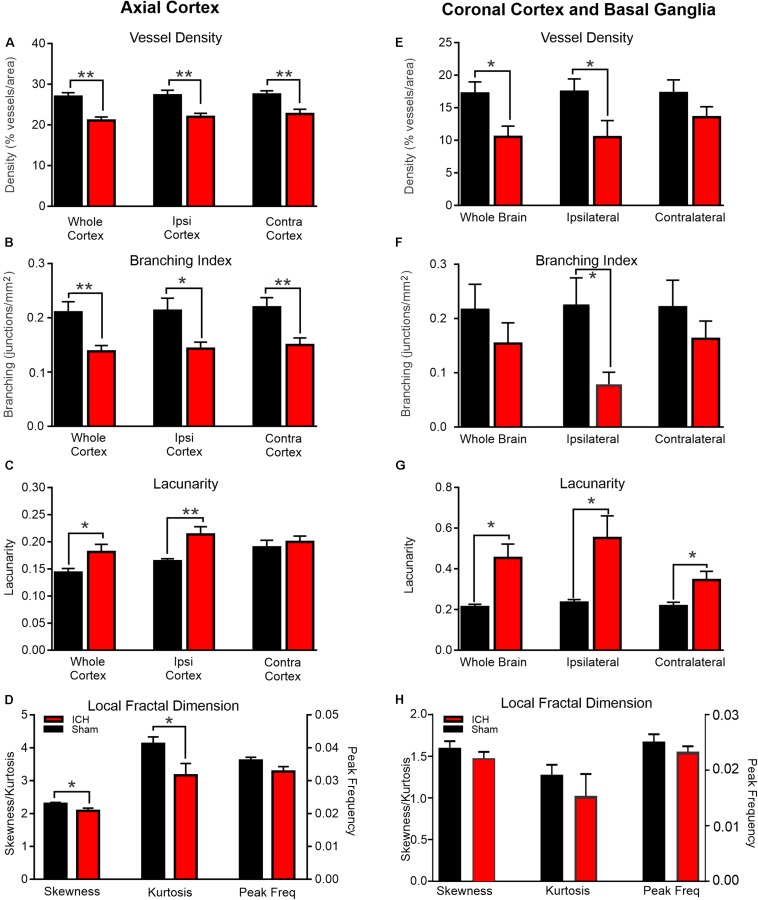
ICH induces modification of vascular features at 24 h post-injury. Axial Cortex: Classical vascular analysis of the entire axial cortex, ipsilateral (Ipsi) and contralateral (Contra) cortical hemispheres resulted in significant reductions in vessel density **(A)** and branching index **(B)** after ICH compared to shams. There was a concomitant increase in lacunarity **(C)** but not in the contralateral hemisphere. Fractal derived vascular complexity of the ipsilateral cortex using local fractal dimension histograms (LFD) **(D)** exhibited significant decrements in skewness and kurtosis between shams and ICH animals. Coronal Cortex and Basal Ganglia: Classical vascular analysis of coronal vessel painted sections at the level of the ICH lesion revealed significant reductions in vessel density **(E)** and reduced branching indices **(F)**. There was a dramatic increase in lacunarity **(G)**. Quantitative analysis of the LFD histograms from the ipsilateral cortex and basal ganglia exhibited no significant changes in skewness, kurtosis or peak frequency **(H)** in ICH vehicle compared to sham vehicle animals (*t*-test **p < 0.03, *p < 0.05).

The morphological features of cortical vessels were derived from the whole axial cortical surface cortex, the ipsilateral (injection side) and contralateral (intact) cortex. Whole brain cortical (both hemispheres) vessel density of Sham + Vehicle animals was 26.94 ± 0.98 compared to 21.09 ± 0.85 in ICH + Vehicle rats (Figure 2A). This reduction in vessel density was significantly decreased by 21.7% (*p* < 0.01) in ICH + Vehicle compared to Sham + Vehicle rats. Similarly, vessel density of ipsilateral axial cortex in ICH + Vehicle rats was significantly decreased by 19.4% (*p* < 0.01) compared to Sham + Vehicle, with Sham + Vehicle having a vessel density of 27.30 ± 1.21 compared to 21.99 ± 0.87 in the ICH + Vehicle animals. Interestingly, contralateral axial cortical vessel density was also significantly decreased by 17.4% (*p* < 0.01) in ICH + Vehicle, where Sham + Vehicle animals had a vessel density of 27.47 ± 0.92 in the contralateral axial cortex compared to 22.69 ± 1.17 in ICH + Vehicle animals.

However, cortical vascular branching (branching index; junctions/mm^2^) followed a similar pattern where ICH + Vehicle animals exhibited significant decreases of 34.2% (*p* < 0.01), 32.8% (*p* < 0.03), 31.6% (*p* < 0.01), respectively across the whole brain, in the ipsilateral hemisphere and in contralateral hemisphere (Figure 2B). As would be expected, lacunarity features were also increased with decreasing vessel density (Figure 2C). In the whole cortex and in the ipsilateral cortex, lacunarity was significantly increased in ICH + Vehicle animals by 21% (*p* < 0.05) and by 23% (*p* < 0.01) compared to Sham + Vehicle rats, respectively. Unexpectedly, the contralateral axial cortex of ICH + Vehicle rats did not exhibit increased lacunarity compared to Sham + Vehicle animals, despite reductions in vessel density and branching indices. Quantitative measurement of the total vessel length of the whole, ipsilateral and contralateral axial cortical vessels revealed no significant differences between the ICH vehicle and sham vehicle groups (data not shown).

#### Coronal Cortex and Basal Ganglia Analysis

Following axial cortical data collection, the brains were cut through the ICH lesion resulting in an anterior and posterior portion of the brain. Wide field fluorescent microscopic images were acquired from the anterior face of the vessel painted brains (Figure 1B). Ipsilateral coronal hemispheric analysis excluded the hematoma. The rationale for these coronal analyses was that it could reveal parenchymal vascular alterations in regions that encompassed cortical and deep gray matter structures such as the basal ganglia.

Coronal vessel density measures of the whole coronal slab in ICH + Vehicle rats was significantly decreased by 38.7% (*p* < 0.03) compared to Sham + Vehicle rats (Figure 2E). The vessel density was 17.20 ± 1.78 in Sham + Vehicle rats compared to 10.53 ± 1.65 in the ICH + Vehicle animals. Similarly, vessel density in the ipsilateral coronal hemisphere of ICH + Vehicle animals were significantly decreased by 39.8% (*p* < 0.05) compared to Sham + Vehicle animals. Sham + Vehicle animals had a vessel density of 17.46 ± 1.97 compared to 10.50 ± 2.53 in the ICH + Vehicle animals. The vessel density of contralateral coronal sections had a 21.4% reduction in ICH + Vehicle compared to Sham + Vehicle animals but was not significantly different (*p* = 0.17).

The branching indices showed considerable variance in the coronal sections which is not overly surprising given differences in the vascular networks between cortical and basal ganglia brain regions (Figure 2F). Only the ipsilateral coronal section exhibited a significant (*p* < 0.03) reduction in vascular branching in ICH + Vehicle compared to Sham + Vehicle animals. Coronal total vessel length measures were not significantly different between ICH + Vehicle and Sham + Vehicle animals, similar to axial total vessel lengths (data not shown).

Lacunarity measures from coronal brain slices were significantly increased by 53.2% (*p* < 0.03), 57.4% (*p* < 0.03) and 37.2% (*p* < 0.03) in whole brain, ipsilateral, coronal and contralateral sections respectively (Figure 2G). In whole brain slices, lacunarity in ICH + Vehicle animals was 0.45 ± 0.09 compared to 0.21 ± 0.014 in sham vehicle rats. The lacunarity of ipsilateral coronal sections of ICH + Vehicle rats were reduced compared to Sham + Vehicle animals at 0.55 ± 0.11 and 0.23 ± 0.015, respectively. Even the lacunarity of contralateral coronal sections were reduced in ICH + Vehicle animals compared to Sham + Vehicle animals (ICH: 0.35 ± 0.04; Sham: 0.22 ± 0.02).

#### Fractal Analysis

Global measures of axial vascular complexity also revealed reductions similar to and validating our classical angiographic measures (Figures 1, 2D). Analysis of the ipsilateral axial cortex found that skewness and kurtosis of ICH + Vehicle animals were significantly (*p* < 0.05) reduced by 9.37 and 23.2% respectively compared to Sham + Vehicle animals. Peak frequency of the local frequency dimension (LFD) histograms which is a measure of the number of vessels was also reduced by 10% in ICH + Vehicle compared to Sham + Vehicle animals but did not reach significance (*p* = 0.09).

Skewness, kurtosis and peak frequency of the LFD histograms of the ipsilateral coronal basal ganglia and cortex showed no significant differences (Figure 2H). ICH resulted in reduced kurtosis and peak frequency of the LFD histograms of ipsilateral coronal basal ganglia and cortex by 46.9% (*p* = 0.42) and 8.2% (*p* = 0.28) respectively in ICH + Vehicle compared to Sham + Vehicle animals. In the automated fractal analyses we were unable to exclude the hematoma from the analysis and likely contributed to the non-significant findings.

In summary, vascular features of the cortex were predominately reduced with the induction of ICH compared to Sham + Vehicle animals. Both axial and coronal assessments yielded similar reductions, further validating our analyses. Interestingly, ICH in many measures also resulted in reductions in contralateral vessel parameters at 24 h after injury. Acutely, other studies of acquired neurological injury have reported similar contralateral effects on the cerebrovasculature.

### Effects of Gleevec on the Vasculature

#### Axial Cortex Analysis

Gleevec (Imantinib) has been reported to be anti-inflammatory and alter the structure of the vasculature following ICH (Soverini et al., 2018; Su et al., 2008). We undertook a more direct measure of the vasculature after Gleevec treatment on vascular features for the ipsilateral hemisphere. For these analyses our comparisons were made between Sham + Vehicle, Sham + Gleevec, ICH + Vehicle and ICH + Gleevec.

**FIGURE 3:**
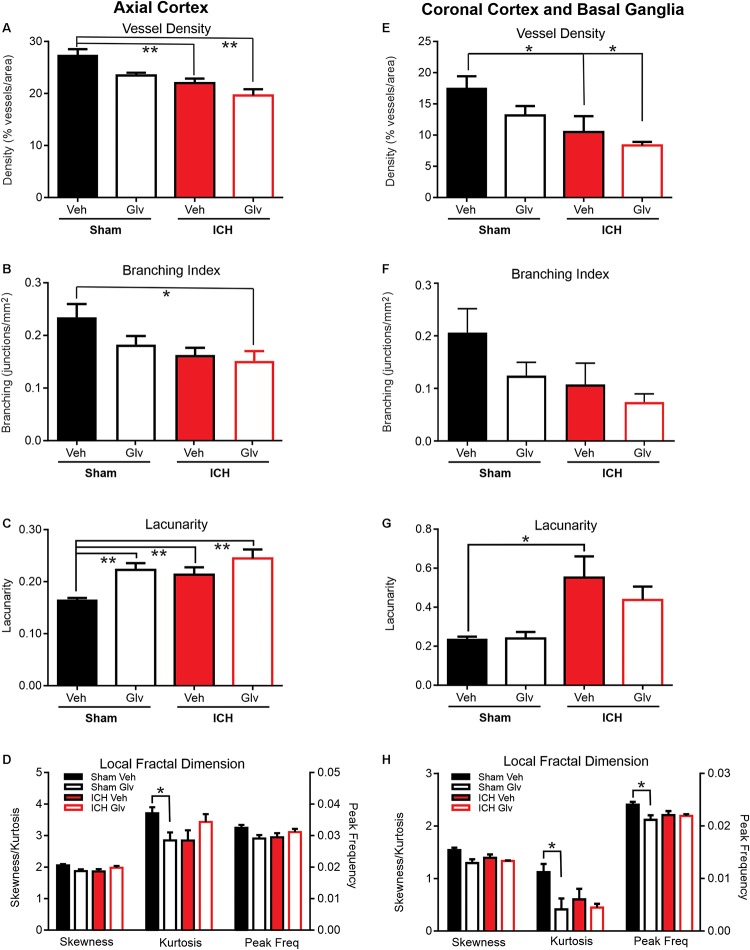
Gleevec treatment does not improve ICH parenchymal vascular measures. Axial Cortex: Ipsilateral cortical vascular analysis revealed no significant changes in vessel density **(A)** or branching index **(B)** between Vehicle nor ICH Gleevec treated rats. However, significant reductions in vessel density were observed between sham vehicle and ICH + Vehicle and ICH + Gleevec rats. Vascular branching was significantly reduced between Sham + Vehicle and ICH + Gleevec rats. Lacunarity **(C)** was only increased in all groups relative to sham vehicle treated rats. Quantitative analysis of the distribution of LFD histograms **(D)** exhibited no significant reductions in skewness, and peak frequency in sham + Gleevec compared to sham + Vehicle animals. Kurtosis was significantly reduced between sham groups. Coronal Cortex and Basal Ganglia: Classical vascular analysis of ipsilateral cortex and basal ganglia revealed a progressively significant decrement in vessel density **(E)** across all groups. *Post-hoc* testing found significant differences between Sham + Vehicle and ICH groups (Vehicle, Gleevec). Branching index **(F)** and lacunarity **(G)** in sham nor ICH animals were not altered, except for a significant increase in lacunarity between sham + Vehicle and ICH + Vehicle rats. The distribution of LFD histograms **(H)** exhibited significant decreases in skewness, kurtosis and peak frequency across groups (*p* < 0.05) but *post-hoc* testing observed significant decreases only in sham groups. (one way Anova with Tukey’s multiple comparisons, **p < 0.03, *p < 0.05).

The density of the axial cerebrovasculature was significantly reduced in ICH + Vehicle and ICH + Gleevec rats compared to Sham + Vehicle (ANOVA, *p* = 0.002, *post hoc* multiple comparisons *p* < 0.01) (Figure 3A). The reduction between Sham + Vehicle compared to ICH + Gleevc was 28.2%. Identical reductions were observed in the branching index (ANOVA, *p* = 0.005, *post hoc* multiple comparisons *p* < 0.01) with a 35.6% reduction in junctions between Sham + Vehicle and ICH + Gleevec groups (Figure 3B). As would be expected with reduced vessel density, lacunarity was significantly increased in Sham + Gleevec, ICH + Vehicle and ICH + Gleevec compared to Sham + Vehicle rats (ANOVA, *p* = 0.002, *post hoc* multiple comparisons *p* < 0.01) (Figure 3C). Lacunarity was significantly increased in Sham + Gleevec by 49.2% (*p* < 0.01) compared to Sham + Vehicle animals. Thus, classical vascular measures of the axial cortex did not show improvement in Gleevec treated ICH animals with treatment at 24 h after ICH induction.

Alternatively, we also undertook a less conservative statistical analysis of just Sham + Vehicle and Sham + Gleevec groups and their vessel characteristics (Supplementary Figure S1) and found that lacunarity was increased significantly (*p* < 0.001) in Sham + Gleevec rats compared to Sham + Vehicle (Supplementary Figure S1C). No significant differences were observed in ICH rats between ICH + Vehicle and ICH + Gleevec groups. Fractal measures were significantly decreased in Sham + Vehicle and Sham + Gleevec rats (Supplementary Figure S1D) but no differences were observed between ICH + Vehicle and ICH + Gleevec groups.

#### Coronal Cortex and Basal Ganglia Analysis

We further investigated whether Gleevec had any effect on parenchymal vascular parameters in coronal brain samples (Figure 1B). Specifically, we examined vessels in the ipsilateral hemisphere (injured) that included the basal ganglia (peri-hematoma, but excluding the hematoma) and adjacent cortex.

Similar to our axial cortical findings (Figure 3A) we observed a significant reduction in vessel density (ANOVA, *p* = 0.002, Tukey’s *post hoc* multiple comparisons *p* < 0.05) (Figure 3E). In ICH + Vehicle and ICH + Gleevec rats there were significant reductions compared to Sham + Vehicle rats, 39.8 and 57.3%, respectively. Total vessel length was unchanged with no differences between groups (data not shown). Branching index trended toward a reduction, like vessel density (ANOVA, *p* = 0.08) (Figure 3F). Again, the largest reductions were in the ICH + Gleevec rats. Lacunarity was increased in ICH animals where a significant increase in lacunarity was observed in ICH + Vehicle rats compared to Sham + Vehicle (ANOVA, *p* = 0.02, Tukey’s *post hoc* multiple comparisons *p* < 0.05) (Figure 3G). Coronal analyses further substantiated a lack of Gleevec in mitigating the acute vascular consequences of ICH on basal ganglia and cortical vessels.

A similar less conservative statistical approach was applied to the coronal cortex and basal ganglia vascular measures (Supplementary Figure S2). No significant differences between Sham + Vehicle and Sham + Gleevec nor ICH + Vehicle and ICH + Gleevec groups were reported for classical vessel features. However, we found fractal measures were significantly decreased in Sham + Vehicle and Sham + Gleevec rats (Supplementary Figure S2D) with no differences observed between ICH + Vehicle and ICH + Gleevec groups. Again, the ANOVA and the *t*-tests for group differences essentially reported similar results.

#### Fractal Analysis

In the axial hemisphere fractal measures, skewness, kurtosis and peak frequency of the local fractal dimensions, were determined. Analysis of the ipsilateral axial cortex found that skewness, kurtosis and peak frequency were significantly reduced (*p* < 0.05) (Figure 3D). In the axial cortex Gleevec caused significant reductions (*p* < 0.03) in both skewness and kurtosis of the LFD histograms by 8.6 and 23.1% respectively in Sham + Gleevec compared to Sham + Vehicle animals (Figure 3D). Peak frequency of the local frequency dimension (LFD) histograms, which is a measure of the number of vessels, was also reduced by 10.3% in ICH + Vehicle compared to Sham + Vehicle animals but did not reach significance (*p* = 0.09). Gleevec resulted in no significant differences in fractal measures between the ICH groups (ICH + Vehicle vs. ICH + Gleevec).

Analysis of skewness, kurtosis and peak frequency of the LFD histograms of the ipsilateral coronal basal ganglia and cortex found significant differences when compared across all groups (Figure 3H) (*p* < 0.05). *Post hoc* analysis of ipsilateral coronal basal ganglia and cortical regions showed that Gleevec resulted in significant reductions (*p* < 0.03; *t*-test) in skewness, kurtosis and peak frequency of the LFD histograms by 15.9, 63.2, and 12.0% respectively in Sham + Gleevec compared to Sham + Vehicle animals. Gleevec resulted in no differences in fractal measures between the ICH groups (ICH + Vehicle vs. ICH + Gleevec), although post-hoc analyses observed significant changes between the sham groups (^∗^*p* < 0.05). These findings match those of the ipsilateral axial cortex (Figure 3D).

The Gleevec fractal results suggest possible alterations in ipsilateral vascular complexity compared to otherwise intact animals (Shams) at 24 h after ICH. These reductions in vascular complexity could play a role in the reported cerebral edema caused by Gleevec in patients with CML4 (Soverini et al., 2018).

### Confocal Vascular Features

**FIGURE 4:**
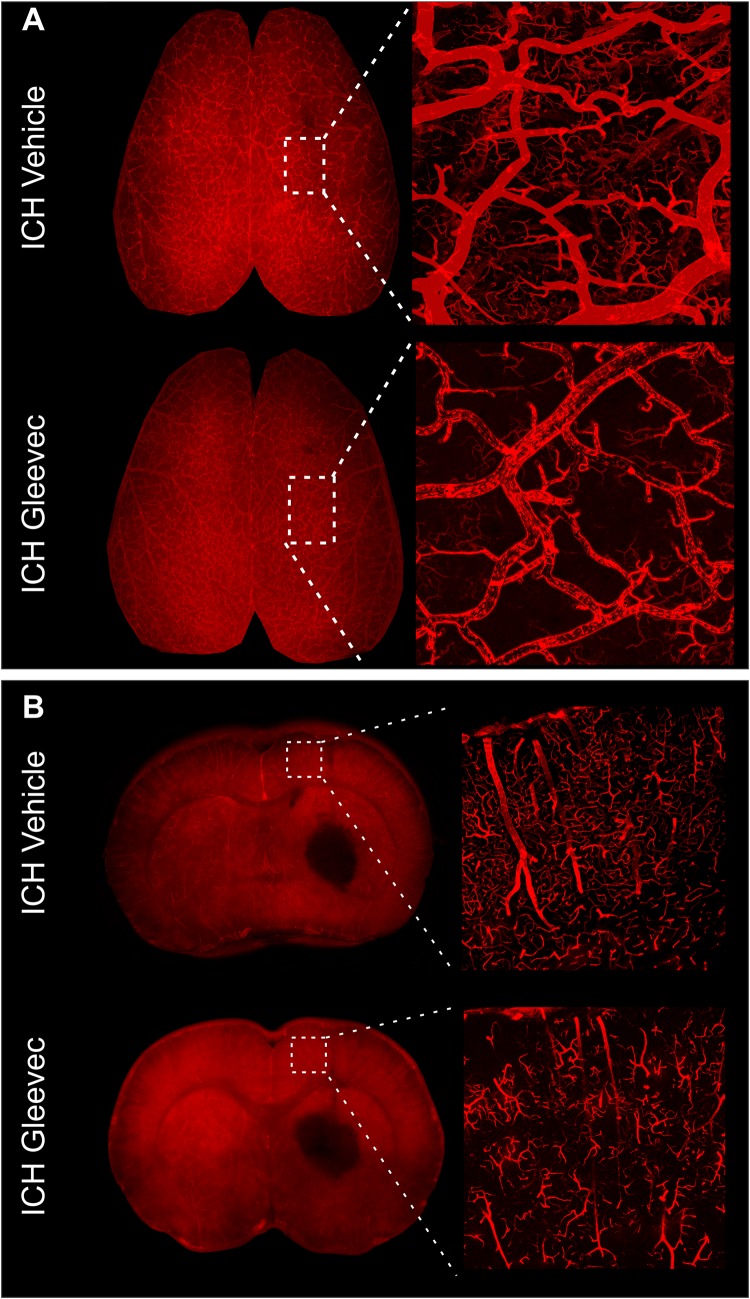
Gleevec treatment does not improve the morphology of the cortical vasculature. **(A)** Vessel painted brains from the axial surface were imaged using confocal microscopy and then subjected to classical vascular analysis. ICH vehicle and ICH Gleevec animals exhibited uniform staining of large, intermediate and small vessels within the cortex. Note the apparent loss of fine branching vessels in the ICH + Gleevec rats. **(B)** In coronal sections from vessel painted brains, the cortical vessels descending into the cortex exhibited a dense plexus of vessels in ICH Vehicle treated rats. In contrast, ICH Gleevec treated rats showed a reduced vascular plexus with fragmented vessels and regions with overt loss of vasculature (see Figure 5 for quantification).

**FIGURE 5:**
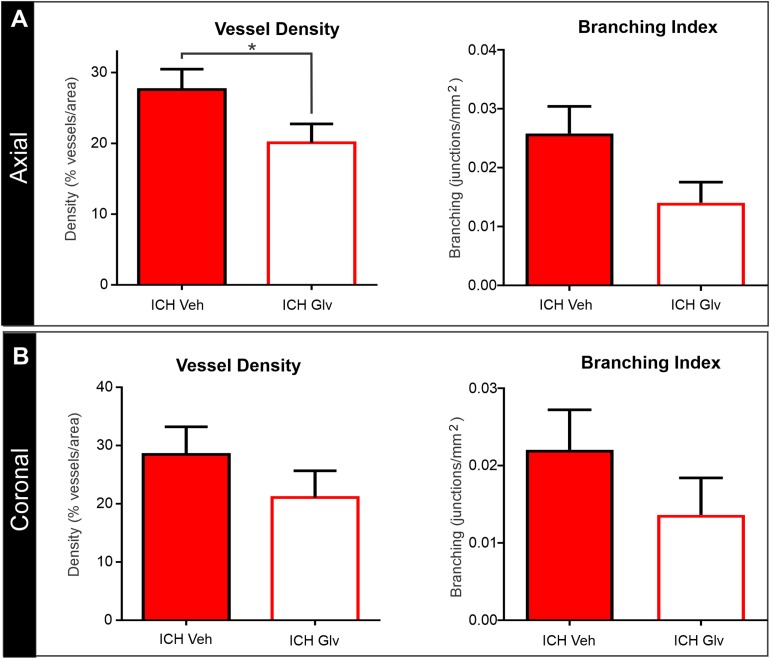
Confocal vascular analysis also demonstrated that Gleevec treatment does not improve vascular density. **(A)** Quantitative assessment of axial cortical vascular features adjacent to the ICH injection site, revealed significant decrements in vascular density and trending decreases in vessel branching after Gleevec treatment. **(B)** Vascular features from cortical sections from coronal tissues also showed decreased vascular density and branching but did not reach significance. Note that both cortical assessments (axial vs. coronal) had similar relative decreases in vascular features (see Figure 4 for images) (*p < 0.05).

The effects of Gleevec on the cerebral vasculature were assessed from axial and coronal confocal images derived from vessel painted brains (Figure 4). All confocal images were acquired adjacent to the site of injection. As can be readily appreciated by visual inspection, Gleevec resulted in reductions in vascular density in ICH rats compared to ICH + Vehicle treated rats (Figures 4A,B). Vessel density was significantly reduced (*p* = 0.05) in ICH + Gleevec compared to ICH + Vehicle rats in axial cortex (Figure 5A). Branching index was also reduced with a trend to significance (*p* = 0.052) as was mean vessel length (*p* = 0.06) but lacunarity was significantly increased in ICH + Gleevec treated rats compared to ICH + Vehicle controls (*p* = 0.03). In the coronal cortex, no significant differences in vessel density (*p* = 0.14), branching index (*p* = 0.13), total vessel length (*p* = 0.09) nor lacunarity (*p* = 0.06) were observed, although some features were trending (Figure 5B).

**FIGURE 6:**
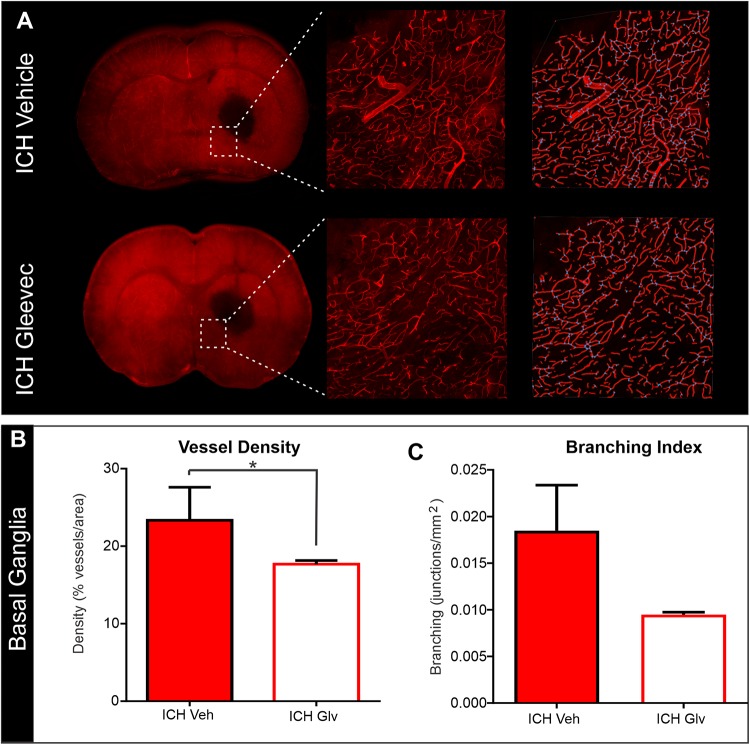
Confocal analysis of the basal ganglia vasculature revealed no improvement with Gleevec treatment. **(A)** The basal ganglia vasculature was assessed for morphological characteristics adjacent to the ICH site. As in the cortex, there was reduced vascular density with loss of fine vascular structures, including branch points. Far right pane illustrates the analysis that derived vessel density (red) and branching (blue dots). **(B)** Quantification of vascular metrics confirmed the visual observations with significantly reduced vascular density (*p* = 0.05) and trending decreases in **(C)** vessel branching (*p* = 0.09) in Gleevec treated compared to Vehicle treated rats.

We also examined vascular features in the basal ganglia adjacent to the hematoma (Figure 6). As can be seen in the confocal microscopy images there was an overt reduction in vessel density in Gleevec treated rats (Figure 6A). Quantification demonstrated a significant decrease in vessel density (*p* = 0.05) in ICH + Gleevec compared to ICH + vehicle treated rats (Figure 6B). Lacunarity in the ICH + Gleevec rats was also significantly increased (*p* = 0.04) compared to ICH + Vehicle controls. No significant differences were found in branching index (*p* = 0.09) nor total vessel length (*p* = 0.70) (data not shown).

### Magnetic Resonance Imaging (MRI)

**FIGURE 7:**
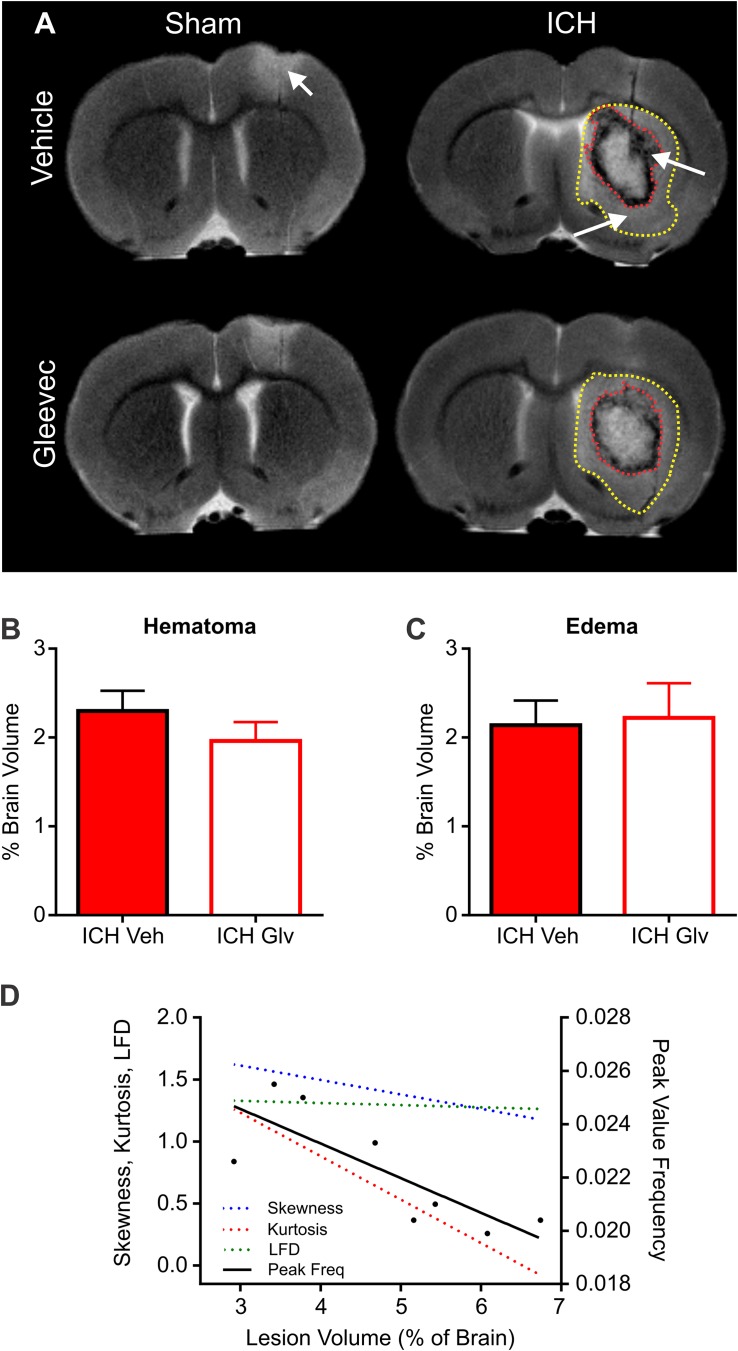
Lesion and hemorrhage volumes were not modified by Gleevec treatment 24 h after ICH. **(A)** Representative MR images from Vehicle and Gleevec treatment rats. Shams exhibited cortical edema (white arrow) at the site of needle insertion. In ICH animals there were hypo- and hyperintense regions visible in the basal ganglia, consistent with blood (red dotted line) and edema (yellow dotted line), respectively (white arrows). **(B)** Subcortical hematoma volumes were not significantly altered between shams and Gleevec treated rats. **(C)** Volume of subcortical edema volumes was not significantly different following Gleevec treatment. **(D)** Correlations of total ICH lesion (blood + edema) volumes compared to ipsilateral fractal properties of ICH vehicle treated rat’s revealed significant correlations. The lesion volume was strongly correlated to fractal skewness (*R*^2^ = 0.6725, *p* = 0.0127), kurtosis (*R*^2^ = 0.6665, *p* = 0.0134), and peak frequency (*R*^2^ = 0.6290, *p* = 0.0189), but not for LFD (*R*^2^ = 0.1194, *p* = 0.4019). See Table 1 for additional correlations.

High resolution *ex vivo* MRI was undertaken to assess hematoma (SWI) and edema (T2WI) volumes (T2WI) (Figure 7). Hematoma volumes were outlined from ICH + Vehicle and ICH + Gleevec rats as shown in Figure 7A (red dotted line). There were no statistical differences for hematoma volumes between the two groups (Figure 7B), where hematoma volumes for ICH + Vehicle rats were 2.30 ± 0.23% of brain volumes and ICH + Gleevec rats had 1.96 ± 0.21% (*p* = 0.31). Similarly, for edema volumes there were no significant differences between groups (Figure 7C). Edema volumes corrected for brain volumes were 2.14 ± 0.28% and 2.22 ± 0.38% in ICH + Vehicle and ICH + Gleevec rats, respectively.

To assess relationship between vascular features and MRI lesion (hematoma + edema) volumes, we correlated vascular complexity measures (skewness, kurtosis, peak LFD value, and peak frequency) to total ICH lesion volumes (Figure 7D). In ICH + Vehicle treated rats, lesion volume (right hemisphere) was significantly correlated to skewness (*R*^2^ = 0.6725, *p* = 0.0127), kurtosis (*R*^2^ = 0.6665, *p* = 0.0134), and peak frequency (*R*^2^ = 0.6290, *p* = 0.0189), but not for maximum LFD (*R*^2^ = 0.1194, *p* = 0.4019) (Figure 7D). We did not observe any such correlations in ICH + Gleevec treated rats (Table 1). Table 1 outlines a number of additional correlations that were undertaken with respect to whole brain and lesion volumes (cortical vs. subcortical). Interestingly, axial cortical measures of vascular density also did not yield any significant correlations to MRI derived volumes (Table 1).

**TABLE 1:** Summary of correlations between MRI derived lesion volumes and fractal vascular features where strong correlations (green) are color coded, as are *p*-values.

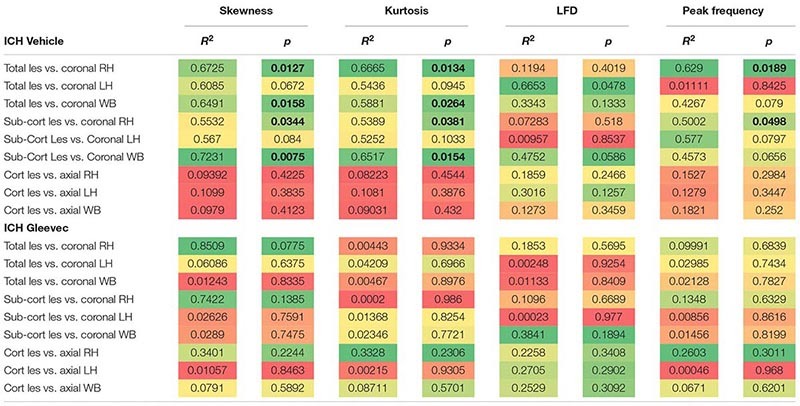

**The red to green spectrum indicates the strength of the correlation, where red is poor and green a strong correlation. Lesion = total lesion volume comprised of hematoma + edema (see Figure 7). Total Les, total lesion volume; Sub-Cort, sub-cortical; Cort, cortical; RH, right hemisphere (ipsilateral); LH, left hemisphere (contralateral); WB, whole brain. Bolded values indicate statistical significance (*p* < 0.05).**

In summary, at 24 h post-ICH there were no significant differences in hematoma, edema nor total lesion volumes as derived from MRI. However, MRI volumes did reflect alterations in vascular complexity but only in ICH vehicle rats. These results further suggest that Gleevec treatment alters vascular phenotype acutely after ICH.

## Discussion

A comprehensive examination of the morphological vascular features after ICH is lacking and prior to evaluation of putative neuroprotective compounds a more complete understanding of how ICH modifies the cerebrovasculature is required. Toward filling this knowledge gap, we now report that ICH resulted in decrements of vascular features acutely following ICH. Specifically, the novel findings we report are, (a) the application of our unique vessel painting methodology to the ICH model, (b) significant reductions in classical vessel features including vessel density and branching, (c) these reductions in vascular morphology are present even distant from the ICH lesion (i.e., cortex, contralateral brain tissues), and (d) measures of vessel complexity using fractal analyses also report decrements. In addition, we used Gleevec to restore or blunt these vessel morphological decrements. Gleevec did not result in improvements but resulted additional vascular reductions in the cortex and in the basal ganglia. Thus, ICH leads to loss of vessels and morphology at 1 day post-injury and Gleevec induces a further exacerbation of vascular density loss.

The effects of ICH on cerebral vessels and blood flow in animal models and in human subjects have been previously described (Zazulia et al., 2001; Xie et al., 2012) and detailed in a recent review (Keep et al., 2014). Most studies investigating the vascular effects of ICH focused on the components of the neurovascular unit (NVU), endothelia, astrocytes and pericytes, with an eye toward blood brain barrier (BBB) breakdown. The disruption of the BBB by ICH leads to numerous downstream (secondary) events including increased bleeding, neuroinflammation and edema formation (Yang et al., 1994; Chen-Roetling et al., 2015; Leclerc et al., 2018; Xu et al., 2019). An 11% reduction in vessel density and volume was reported adjacent to the ICH lesion using high resolution micro-computed tomography (Xie et al., 2012). Functionally, Zazuila and colleagues found a 44% decrease in cerebral blood flow adjacent to the ICH clot within 24 h, consistent with our results that showed a decrease in vascular density and edema at the same time point (Zazulia et al., 2001). Thus, whilst preservation of the BBB is important following ICH, a more thorough understanding of how the morphology of vessels are modified by ICH is clearly warranted prior to studies targeted to improving the vasculature.

We undertook in the present study a quantitative evaluation of the alterations in vessel morphology acutely after ICH. We report a dramatic ∼20% reduction in vessel density in the whole cortex as well as the ipsilateral and contralateral cortices. The observed reduction was distant from the ICH site of injury and in the basal ganglia we observed a 39% decrement in vascular density. Other vascular metrics such as vessel branching were similarly reduced at 24 h post-ICH induction. The marked reduction in vessel density within the basal ganglia would be expected based on the reported mass effects (Brouwers et al., 2014), ferroptosis (Wan et al., 2019) and associated redox activities known to occur after ICH (Chen-Roetling et al., 2015; reviewed in Keep et al., 2014). What was surprising were the global (ipsi- vs. contralateral) cortical reductions in vessel features (density, branching, etc.). In the acute stages of ICH (<24 h) numerous mechanisms could contribute to distant modifications of brain vessels (Aronowski and Zhao, 2011; Ren et al., 2014; Schlunk and Greenberg, 2015). Indeed, we have reported on a yet unidentified diffusible factor after ICH that can alter the structure of vessels distant from the injury site that likely modifies vessel morphology as well as function (Pearce et al., 2016).

We suggest that a combination of factors contribute to the reductions in vessel features we observed following ICH. Firstly, as we and others have reported there is a significant edematous response within the basal ganglia surrounding the perihematomal region (Yang et al., 1994; Belayev et al., 2003; Xu et al., 2019). This robust disruption of the vasculature and subsequent BBB leads to increased vascular permeability and edema formation (see Figure 7) in the collagenase model of ICH. Secondly, the deposition of red blood cells and subsequent lysis increase iron and thrombin at the ICH lesion site (Hua et al., 2007; Wan et al., 2019). This results in a neuroinflammatory cascade that certainly further exacerbates existing vessels and might play a significant role in subsequent lesion expansion. A number of key proteins involved in inflammation, necrosis and apoptosis, have been shown to be upregulated acutely after ICH induction (Ren et al., 2014). Thirdly, a number of endothelial junction proteins and regulatory mechanisms that act synergistically to maintain vascular integrity are known to be impacted by ICH and have been comprehensively reviewed recently (Keep et al., 2018). Thus, a wide range of cellular and molecular mechanisms could directly impact vessel morphology both at the ICH lesion and surrounding tissues and also at distant cortical structures. Further research is needed to clarify the mechanisms that impact the cerebrovascular network after ICH.

A range of interventions have been applied to mitigate the neurobiological effects of ICH and its subsequent poor outcomes (Hatakeyama et al., 2013; Chen-Roetling et al., 2015; Yang et al., 2016; Leclerc et al., 2018; Xu et al., 2019). However, most of the interventions for ICH to date have been neuro-centric, that is, focused on neuroprotection. While protection of cellular elements such as neurons is important, an intact vascular network is critical for neuroprotection with the delivery of nutrients and removal of waste products. There is increasing recognition that platelet-derived growth factor (PDGF; a sub-family of tyrosine kinase receptors) and its various ligands and receptors are critical for the health and stability of endothelial cells and pericytes (Kazlauskas, 2017; Heldin et al., 2018). PDGFs and their isoforms have also provided new treatment options, particularly for manipulating angiogenesis in cancer (Paulsson et al., 2014; Heldin et al., 2018). Foundational studies developed therapeutic interventions targeting tyrosine kinase in vessels to inhibit tumor growth by targeting angiogenic processes, including those associated with PDGFs (Soverini et al., 2018).

As noted above, we and others have targeted PDGF receptors as a therapeutic option for ICH using Gleevec (imatinib mesylate) (Ma et al., 2011; Pearce et al., 2016; Yang et al., 2016). We found that Gleevec treatment following ICH blunts the phenotypic transformation of smooth muscle surrounding arteries (Pearce et al., 2016), reduced BBB disruption leading to improved outcomes (Ma et al., 2011) and mitigated plasmin induced vascular inflammation (Yang et al., 2016). Many of the beneficial effects of Gleevec were found to occur acutely (<24 h) with long term improvements but the direct effects on vascular morphology have not been reported upon.

While arterial smooth muscle phenotypes are rescued by Gleevec, likely through modulation of neuroinflammation, there have been no direct studies examining the vascular morphology after Gleevec treatment. We sought to examine the effectiveness of Gleevec treatment delivered within 1 h after ICH in preventing the vascular alterations observed after lesion induction. Much to our surprise, in general, we found no improvements in cortical vascular morphology after Gleevec treatment 24 h after ICH. ICH + Gleevec rats had significant reductions in vascular measures compared to Sham + Vehicle treated rats, but statistical analyses of group measures found no statistically significant differences between ICH + Gleevec and ICH + Vehicle rats (see Figure 3). When comparisons were made for both axial and coronal vessel painted brain sections of the cortex, a modest but significant reduction in vessel density was reported (Figure 5). Despite statistically insignificant reductions in vascular parameters there appeared to be an overall trend for continued decrements following ICH + Gleevec treatment compared to ICH + Vehicle treated rats in both visual assessments and quantitative findings (Figures 3–5). The perihematomal region also had statistically significant reductions in vessel density but not vessel junctions (Figure 6). Gleevec also did not modify edema nor blood volumes at 24 h after ICH. Overall, Gleevec did not directly improve vascular morphological features at 24 h after treatment. In fact, in some measures there appeared to be a modest further reduction in vascular elements.

Initially, we were surprised by the lack and perhaps enhanced detrimental effects of Gleevec. There are several factors that could be responsible for the lack of therapeutic effectiveness on the vessels. One clear possibility is that we assessed vessel morphology during the early acute phase of the ICH injury, a period during which many molecular processes are being activated (Ren et al., 2014). The prevention of acute changes in vascular smooth muscle we previously reported appeared to be due in part to probable reductions in neuroinflammatory processes (Yang et al., 2016). Further, in CD163−/− mice there was a decreased acute (3 days) inflammatory response to ICH (Leclerc et al., 2018). Similarly, heme oxygenase 1 (HO-1) activation and overexpression in astrocytes improved BBB disruption after ICH, acutely (Chen-Roetling et al., 2015). HO-1 has also been shown to have a vasoprotective effect on vessels in the retina (He et al., 2014). Thus, inflammatory mediators in conjunction with coagulopathic mechanisms that are known to be active during ICH may acutely inhibit vascular remodeling necessary for long-term neurological improvements (Ren et al., 2014; Liu et al., 2018). It is also possible that Gleevec treatment modified vessel staining while using the vessel painting method. However, we do not believe that Gleevec resulted in adverse vessel labeling as we utilized a potent vasodilator, sodium nitroprusside, at the time of perfusion (see methods). Further, several reports have reported a suppression of vascular contractility following Gleevec treatment (Gur et al., 2013; Tang, 2015) including our own findings (Pearce et al., 2016).

PDGF is well known to have angiogenic activities and differential roles for its isoforms have been noted including recruitment of pericytes (Zhang et al., 2009). Tan and colleagues reported increased angiogenesis around the hematoma at 7–14 days after ICH likely via a vascular endothelial growth factor (VEGF) mechanisms (Tang et al., 2007) and confirmed in subsequent reports (Zhou et al., 2012). This vascular repair timeline is very similar to what we have reported in traumatic brain injury, albeit via a Wnt/β-catenin mechanism (Salehi et al., 2018a). In stroke, Gleevec protected the BBB, as well as “normalized” vessels within the brain early (1 h) post-injury (Su et al., 2008). It is likely that in our acute ICH study we did not observe any angiogenic potential due to Gleevec treatment, as this repair mechanism under baseline conditions appears to take several days. In future studies we will evaluate the previously reported spontaneous angiogenesis and whether this might be accelerated by Gleevec treatment.

Gleevec and subsequent generation tyrosine kinase inhibitors are known to act at BCR-ABL and c-KIT sites that can regulate the response of the cerebrovasculature following brain injury (see Rossari et al., 2018, review). However, adverse vascular events have been reported in patients treated with Gleevec or other related tyrosine kinase inhibitors (Pasvolsky et al., 2015). It is intriguing to speculate that the early lack of effect on vascular morphology seen in our study could be related to downstream effects of Gleevec on BCR-ABL on Wnt/β-catenin cascades (Cilloni and Saglio, 2012) which we have previously reported is involved in late vascular remodeling following traumatic brain injury (Salehi et al., 2018a).

Neuroimaging using clinically relevant modalities such as computed tomography and magnetic resonance imaging (MRI) are potent methods for non-invasively assessing the degree of hemorrhage and edema in patients and in preclinical models (Belayev et al., 2003; Goh et al., 2015; Heit et al., 2017). In our present study we also acquired T2 weighted MRI for edema and susceptibility weighted (SWI) MRI for blood content to assess if there was a correlation between vessel features and clinically relevant MRI. No correlations were observed between classical vascular measures (density, etc.) and lesion volumes. However, as noted in Table 1 and in Figure 7 there were several strong correlations between total lesion volumes and vessel features derived from fractal measures of vascular complexity in ICH + Vehicle treated rats. We observed that vascular complexity was reduced after ICH but not in ICH + Gleevec rats. For example, as lesion volume (edema + hematoma) increases, vascular complexity was reduced (Figure 7D, peak frequency reductions). Similar, reductions in vascular complexity using fractal analysis was observed at 1d post-traumatic brain injury (Obenaus et al., 2017). Interestingly, Gleevec treatment resulted in a loss of significant correlations between lesion characteristics and fractal features of complexity (Table 1). While it is difficult to ascertain what the underlying mechanism(s) might be for Gleevec in modulating vascular complexity, it is clear that many of the smaller vessels appear to be lost after treatment (see Figure 4). It is possible that the global reduction in vascular complexity due to Gleevec treatment could further disrupt fractal measures leading to loss of correlative measures. Interestingly, fractal analysis of ICH lesions from computed tomography were found to be a good predictor of outcomes (Klis et al., 2018). Thus, MRI-derived hematoma and edema volumes can reflect the relative loss of vascular complexity, but additional research is needed to corroborate these findings.

## Conclusion

In conclusion, this study for the first-time reports on quantitative measures of the vascular network within 24 h after ICH induction using a novel vessel painting methodology (Salehi et al., 2018b). The acute reductions in vessel density (and other features) after ICH were not reversed by treatment with Gleevec. While previous reports have suggested a restorative effect on vessels with Gleevec treatment, these studies did not directly assess vessel morphology but rather constituents of the BBB and/or artery smooth muscle phenotypes (Pearce et al., 2016; Yang et al., 2016). Inhibition of inflammatory mediators appears to restore the neurovascular unit and limit BBB disruption but in our hands Gleevec did not appreciably alter the morphology of the vascular network (Wang and Dore, 2007; Mracsko and Veltkamp, 2014). BBB improvements and PDGF modulation of neuroinflammation are likely independent of vessel morphology. Vascular morphology has at least two important components, (a) number of arteries/capillaries and (b) structure of arteries/capillaries. While loosely related modification in one aspect does not necessarily lead to alterations in the other. Smooth muscle can also upregulate and downregulate expression of membrane receptors and ion channels, without any obvious change in structure. From this perspective, our observed changes in vessels are a somewhat crude assessment of vascular response. While it was not examined in the present study, future studies should examine vascular reactivity and perfusion as they are important in understanding the cellular and molecular underpinnings for the vascular morphological changes we observed. Finally, MRI derived hematoma measures related to vascular complexity in ICH + Vehicle but not ICH + Gleevec treated rats. More research is required to better understand the relationship between the vascular network’s response to ICH and how putative therapies may enhance vessel structure and function. Thus, vascular morphology is an important outcome measure in assessing future therapies for treatment of ICH.

## Data Availability Statement

The datasets generated for this study are available on request to the corresponding author.

## Ethics Statement

The animal study was reviewed and approved by Loma Linda University Institutional Animal Care and Use, Loma Linda University.

## Author Contributions

All authors had full access to all the data in the study and take responsibility for the integrity of the data and the accuracy of the data analysis. MA, MH, JT, WP, JZ, and AO: conceptualization. MA, MH, DN, and AO: investigation. MA, MH, and EH: formal analysis. MA, MH, and AO: writing – original draft. MA, MH, JT, WP, JZ, and AO: writing – review and editing. MA, MH, EH, and AO: visualization. AO: supervision. JZ, JT, WP, and AO: funding acquisition.

## Conflict of Interest

The authors declare that the research was conducted in the absence of any commercial or financial relationships that could be construed as a potential conflict of interest.
