# Supplementary material for: Acute Treatment With Gleevec Does Not Promote Early Vascular Recovery Following Intracerebral Hemorrhage in Adult Male Rats
Source: Front Neurosci. 2020 Feb 4;14:46. doi: 10.3389/fnins.2020.00046 (PMC7010856; doi:10.3389/fnins.2020.00046)
Supplement: Supplementary file 1 [file Table_1.DOCX]

Supplementary Material

## Supplementary Figures


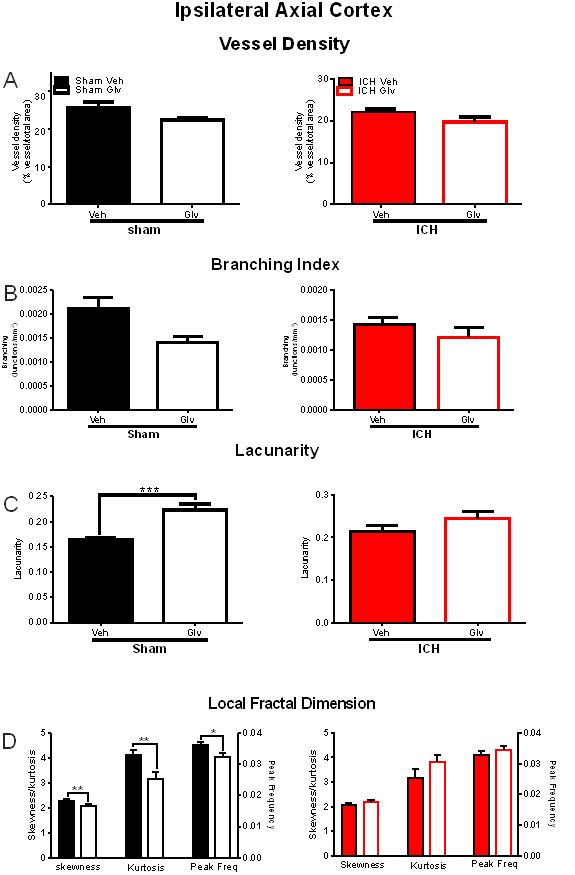


**Supplementary Figure 1.** Gleevec treatment did not improve parenchymal vasculature in the cortex. Ipsilateral cortical vascular analysis revealed no significant changes in vessel density (A) or branching index (B) between Sham+vehicle and Sham+Gleevec (left panels) comparisons nor between ICH+vehicle and ICH+Gleevec rats (right panels). Lacunarity (C) was only increased in between Sham+vehicle and Sham+Gleevec animals but not in ICH+vehicle and ICH+Gleevec rats. Quantitative analysis of the local fractal dimension histograms (D) found significant reductions between Sham+vehicle and Sham+Gleevec rats in skewness, kurtosis and peak frequencies. No significant changes were observed in ICH+vehicle and ICH+Gleevec rats. (t-test ***p<0.01, **p<0.03, *p<0.05)

**
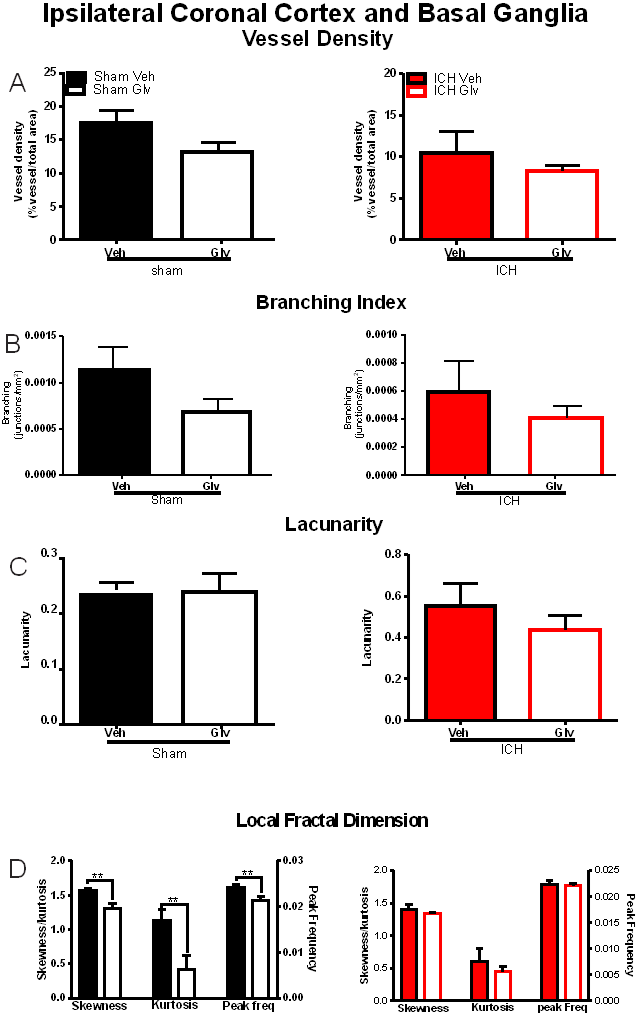
**

**Supplementary Figure 2.** Gleevec treatment did not improve parenchymal vasculature in the coronal cortex and basal ganglia: Classical vascular analysis of ipsilateral cortex and basal ganglia found no significant changes in vessel density (A), branching index (B) or laucnarity between Sham+vehicle and Sham+Gleevec (left panels) comparisons nor between ICH+vehicle and ICH+Gleevec rats (right panels). Quantitative analysis of the local fractal dimension histograms (D) found significant reductions between Sham+vehicle and Sham+Gleevec rats in skewness, kurtosis and peak frequencies. No significant changes were observed in ICH+vehicle and ICH+Gleevec rats (t-test ***p<0.01, **p<0.03, *p<0.05).
